# Pulmonary Infection Caused by *Mycobacterium conceptionense*

**DOI:** 10.3201/eid1801.110251

**Published:** 2012-01

**Authors:** Seon Young Kim, Myung Shin Kim, Ho Eun Chang, Jae-Joon Yim, Jae-Ho Lee, Sang Hoon Song, Kyoung Un Park, Junghan Song, Eui-Chong Kim

**Affiliations:** Seoul National University College of Medicine, Seoul, South Korea (S.Y. Kim, J.-J. Yim, J.-H. Lee, S.H. Song, K.U. Park, J. Song, E.-C. Kim);; Seoul National University Bundang Hospital, Gyeonggi-do, South Korea (M.S. Kim, H.E, Chang, J.-H. Lee, S.H. Song, K.U. Park, J. Song)

**Keywords:** Mycobacterium conceptionense, pulmonary infection, tuf, 16S rDNA, tuberculosis and other mycobacteria, bacteria, South Korea

**To the Editor**: *Mycobacterium conceptionense* was first identified in 2006 from a patient with posttraumatic osteitis (*1*). Since then, 3 more isolates have been recovered from a subcutaneous abscess (*2*), a wound after breast surgery (*3*), and an abscess after a fat injection (*4*). During November 2009 through April 2010, *M*. *conceptionense* was isolated from sputum from 4 patients in 2 tertiary hospitals in South Korea.

Patient 1, a 69-year-old woman, was admitted to Seoul National University Bundang Hospital in 2005 with fever and pleuritic chest pain. She had a long history of recurrent fever and cough. Computed tomography (CT) showed multifocal nodular lung lesions with lymphadenopathy. After 7 days of treatment with cefuroxime and azithromycin, the patient’s fever subsided and radiographic lesions disappeared. She was discharged with negative culture results. After discharge, she had recurrent episodes of fever, and CT showed waxing and waning pulmonary lesions. Nontuberculous mycobacteria (NTM) species were isolated from some sputum cultures: *M. smegmatis* in 2006; *M. avium* in 2007; and *M. intracellulare* in 2008 and 2009. In February and April 2010, her respiratory symptoms and chest CT findings indicated more severe disease, and *M. conceptionense* grew in sputum cultures. After treatment with clarithromycin, rifampin, and ethambutol for 2 months, the patient’s symptoms improved and sputum culture results were negative.

Patient 2, a 70-year-old man with Parkinson disease, was referred to Seoul National University Bundang Hospital in November 2009 for a small nodular lung lesion detected by CT during a medical checkup. He exhibited no pulmonary symptoms. Routine laboratory test results were within normal limits. *M. conceptionense* was isolated from sputum. Clarithromycin was prescribed for 10 days, and the patient remains asymptomatic.

Patient 3, a 70-year-old man with tongue cancer, was admitted to Seoul National University Hospital in March 2010 with exacerbated dyspnea. In November 2009, CT had indicated new nodular lung lesions and chemotherapy had been started. Chest CT in 2010 showed increased size and extent of nodular infiltration, which suggested pulmonary infection rather than cancer metastasis. From 2 sputum samples, 2 isolates of *M. conceptionense* were identified. In addition, *Streptococcus pneumoniae* grew in blood and sputum cultures. Despite treatment with broad-spectrum antimicrobial drugs, the patient died of respiratory failure.

Patient 4, a 53-year-old man, sought care at Seoul National University Hospital in 2008 for chest discomfort. Other than having diabetes mellitus, he had been healthy. Chest CT showed multiple lung nodules. Sputum culture grew *M. tuberculosis*. The patient received isoniazid, rifampin, ethambutol, and levofloxacin for 6 months, during which time sputum cultures were negative. In April 2010, follow-up sputum culture grew *M. conceptionense*. The patient was asymptomatic and followed up without treatment.

Cultures for each patient were conducted at the respective hospitals, where sputum specimens were placed on solid media (Ogawa; Shinyang, Seoul, South Korea) and in liquid media (MGIT 960; Becton Dickinson, Sparks, MD, USA) after decontamination with NaOH. For all 6 specimens, acid-fast bacilli grew 4–7 days after incubation in liquid media.

Molecular identification was conducted at Seoul National University Bundang Hospital, where PCR restriction fragment length polymorphism and multiplex real-time PCR and melting curve analyses were performed as described (*5**,**6*). Each method produced identical results for all but did not support specific identification. PCR restriction fragment length polymorphism profiles and melting peaks for the isolates from patients 1–4 were similar to those of *M. septicum* and *M. fortuitum*. Sequence analyses of the 652-bp fragment of *tuf* and the 527-bp and 1,571-bp fragments of 16S rDNA genes were performed (*7**,**8*). The *tuf* sequences of isolates from patients 1, 3, and 4 showed 100% identity with the *M. conceptionense* type strain, 98.2% homology (11-bp difference) with *M. porcinum*, and 98.1% homology with *M*. *fortuitum*. The *tuf* sequence of the isolate from patient 2 differed by 2 bp from the others. The 16S rDNA sequence of the isolate from patient 1 showed 100% homology with sequences of *M*. *conceptionense* and *M*. *senegalense* and 99.9% (2-bp difference) homology with *M*. *farcinogenes*. Broth microdilution susceptibility tests for isolates from patients 1, 2, and 4 showed susceptibility to amikacin, ciprofloxacin, clarithromycin, and doxycycline but resistance to cefoxitin, sulfamethoxazole, rifampin (MIC >16 μg/mL) and intermediate-resistance to imipenem (MIC 8–16 μg/mL).

According to the American Thoracic Society diagnostic criteria for NTM lung disease (*9*), patient 1 fulfilled all criteria and patient 3 fulfilled the radiographic and microbiological criteria. These findings suggest that *M. conceptionense* can cause lung disease. For the other patients, colonization with *M. conceptionense* is a more plausible explanation (Table).

**Table:** Summary of cases of *Mycobacterium conceptionense* infection*

Patient no.	Age, y/ sex	Underlying illness	Clinical presentation	*M. conceptionense* source	Sequencing results†	Treatment	Outcome	Ref
1	69/F	Chronic lung disease	Chronic cough and recurrent fever; multifocal lung lesion and lymphadenopathy seen on chest CT image	Sputum (2×)	*tuf,* 16S rDNA (1,441 and 458 bp; 100% match)	CLA, RIF, EMB	Improved after 2 mo treatment	This article
2	70/M	Parkinson disease	Asymptomatic; small nodule seen on chest CT image	Sputum (1×)	*tuf*	CLA	Asymptomatic	This article
3	70/M	Tongue cancer	Respiratory failure, *Streptococcus pneumoniae* septicemia; lung lesion on chest CT image	Sputum (2×)	*tuf,* 16S rDNA (400 and 460 bp; 100% match)	CLA, LVX, IPM, AMK, VAN	Died	This article
4	53/M	Lung tuberculosis	Asymptomatic after completion of antituberculosis treatment	Sputum (1×)	*tuf,* 16S rDNA (459 bp; 100% match)	Observation	Asymptomatic	This article
5‡	31/F	Posttraumatic osteitis	Wound liquid outflow 3 mo after treatment for open fracture	Wound liquid, bone tissue biopsy, excised skin tissue	16S rDNA, *soda*, *hsp65*, *recA*, *rpoB*	AMC	Not reported	(*1*)
6‡	43/F	Subcutaneous abscess without trauma	Painful swelling and erythematous ankle; abscess detected by MRI	Abscess aspirate	16S rDNA (1,464 bp)	COT, CLA, DOX, LIN	Improved after 5 mo treatment	(*2*)
7‡	58/F	Breast implant infection	Fever and wound discharge	Wound discharge, surgical drainage	*rpoB*	CIP, AZY, DOX	Unremarkable results at 2-mo follow-up after 18 mo treatment	(*3*)
8	50/F	Face surgery with fat grafting	Erythematous nodules and purulent discharge	Wound discharge	16S rDNA, *rpoB*	AMK, LVX, CFX, CLA, SXT	Recovered after 1 mo treatment	(*4*)

*Ref, reference; CT, computed tomography; CLA, clarithromycin; RIF, rifampin; EMB, ethambutol; LVX, levofloxacin; IPM, imipenem; AMK, amikacin; VAN, vancomycin; AMC, amoxicillin/clavulanic acid; MRI, magnetic resonance imaging COT, cotrimoxazole; DOX, doxycycline; LIN, linezolid; CIP, ciprofloxacin; AZY, azithromycin; CFX, cefoxitin; SXT, sulfamethoxazole/trimethoprim. †Sequences of the isolates were compared with the *tuf* gene and 16S rDNA gene sequence of the type strain CIP 108544^T^ (GenBank accession nos. EU191943.1 and AY859684.1, respectively). ‡Data modified from Thibeaut et al. (*3*).

These 4 recent cases of *M. conceptionense* infection are in accordance with the increasing prevalence of NTM (*10*). Increasing prevalence might be the result of technical advances in NTM identification, including use of liquid media and sequencing, or the result of a local outbreak or contamination event. We consider contamination to be an unlikely cause because specimens were completely separated from each other during collection and testing. Isolates from different patients yielded distinct randomly amplified polymorphic DNA patterns. In conclusion, *M*. *conceptionense* is not a rare NTM species in South Korea and can cause pulmonary disease.
